# On the Efficacy of the Digitalis Purpurea, in Allaying Excessive Action of the Sanguiferous System: Communicated to Dr. Mitchell

**Published:** 1808-05

**Authors:** James S. Stringham

**Affiliations:** New-York


### *On the* Efficacy *of the* Digitalis Purpurea, *in* allaying excessive Action *of the* Sanguiferous System: *Communicated to Dr.* Mitchell *by* James S. Stringham, *M. D. of New-York.*

THE latter end of the month of April last I was called upon to visit a gentleman in this city, who was labouring under a severe attack of pneumonia. When I saw him (which was on the third day of the disease), his face was highly flushed, the tunica albuginea extremely turgid. About two hours before I came he had been seized with delirium; complained of great pain, particularly on the right side, shooting from thence to the scapula; his skin was hot and dry; pulse about 120; respiration laborious, attended with with a short dry cough. The usual remedies had been applied by the physician who first attended him; but they had contributed little to his relief. The obstinacy and violence of the fever made me determine, if possible, to administer some remedy which might, by its first and immediate operation, diminish the action of the arterial system. Of those contained in the materia medica, I formed the most favourable expectations from the digitalis, as I thought that in one case, where it had been taken by mistake to the quantity of three grains, it had, in this way, greatly assisted in the cure of an incipient enteritis, though I, at that time, attributed its good effects not to any action peculiar to the digitalis, but to the great nausea and vomiting it occasioned, which terminated in a profuse sweat. I, consequently, thought that it might properly be administered with a twofold view. First, I hoped that by giving it in small doses, and at short intervals, my patient might be able to retain a sufficient quantity of it on his stomach to act quickly and generally on the system. If I was disappointed here, and should nausea or vomiting be produced, I hoped that these might be succeeded by that evacuation which had proved so salutary in the case just alluded to. Accordingly, at ten o'clock on the same morning in which I was first called, I began by ordering him to take half a grain every three hours, unless he found his stomach or head affected by it. At seven in the evening I visited him again, and found the quickness of pulse greatly diminished; and his nurse remarked to me, that he had voided more urine that day than on any other during his illness. He had had no delirium during the last six hours; nor was that turgidity of the countenance by any means so remarkable as in the morning.

He had now taken two grains of the digitalis. Encouraged by a change so flattering (as the man was of a strong and robust constitution), I determined, by pushing my medicine, to give it a fair trial. I therefore ordered a continuance of it, with the same proviso as formerly. Feeling some anxiety as to the event of my experiment, and not placing the greatest confidence in the attention and punctuality of the nurse, I visited him again at nine. He had had another profuse discharge of urine since I saw him last; and every appearance was so much changed for the better, that I did not hesitate to pronounce him out of danger. His pulse now did not exceed ninety strokes in a minute. I was present at his taking the fifth dose, which, shortly after it was received into the stomach, pro-

 duced duced what he termed an insupportable nausea. This was succeeded by vomiting, which not being very severe, I encouraged by diluents. After this the medicine operated briskly as a cathartic. I continued with him for some time; and, after ordering a discontinuance of the digitalis, I left him composed, and with an incipient moisture upon the skin. The next morning I found that he still continued convalescent. On inquiring whether he had since felt any disagreeable effects from his medicine during the night, he assured me that he had not, except an uncomfortable dergee of sweating. On the third day of my visiting him, I observed that with his cough he had some expectoration. I now gave him the oxymel scilliticum, at the same time paying attention to the state of his bowels. Upon this plan of treatment, in a short time, he so far recovered as to render my further attendance unnecessary.

---

Shortly after the above trial, a school-master in this city consulted me about a fixed and obstinate pain in his breast, which he had experienced at intervals during several years, first occasioned, as he supposed, by a too intense application to writing. It may be proper to remark, by the way, that in this case there was no cough. After taking some blood from him, and opening his bowels, I applied blisters successively to the painful part. To these was also added the use of diaphoretics, and an antiphlogistic regimen, but all without any evidently good effect. I determined, in this case also, to try the virtues of the digitalis. He began with half a grain night and morning, which, after a few days, was increased to a grain. He continued persevering in this plan about a fortnight, when he assured me that he found himself greatly better, and wished to know whether it was necessary to continue the medicine. This I strongly recommended; notwithstanding which, his neglect of remedies (as commonly happens) increasing in proportion as his complaint diminished, the pain again returned with its former severity. I again put him on the same plan, with the same success—as he now assures me that it does not prevent him from prosecuting the duties of his station with ease and satisfaction.

---

I have often thought, that a medicine which would diminish the action of the vascular system, without previously occasioning an increased excitement, was a great desideratum amongst physicians. The cases just now related lated induce me to hope that this want may be, at least in some degree, supplied by the digitalis, as its first effect (so far as I could observe) after its exhibition, was that of rendering the pulse slower. With this circumstance, I also apprehend, are intimately connected that increase in the quantity of urine and subsequent perspiration, which seemed, in so short a time, to place my first patient entirely out of danger. I have ever considered a too rapid circulation of the fluids equally unfavourable to perspiration and secretion with that which is too tardy and languid. When the blood is thus precipitately hurried away, a sufficient time is not given for the separation of its thinner parts, destined to enter either the small and delicate excretories whichopen on the surface, or those still more minute ramifications which constitute the glands.

